# Supplementary material for: A Bayesian network meta-analysis of the primary definitive therapies for locoregionally advanced nasopharyngeal carcinoma: IC+CCRT, CCRT+AC, and CCRT alone
Source: PLoS One. 2022 Mar 18;17(3):e0265551. doi: 10.1371/journal.pone.0265551 (PMC8932567; doi:10.1371/journal.pone.0265551)
Supplement: S1 Fig — Network for 5-year overall survival (A), failure-free survival (B), distant metastasis-free survival (C), and locoregional recurrence-free survival (D). each circular node represents a type of treatment. The dot size is proportional to the total number of patients who received a regimen. Each line represents a head-to-head comparison and the line width is proportional to the number of clinical trials comparing the connected treatment strategies. (DOCX) [file pone.0265551.s002.docx]

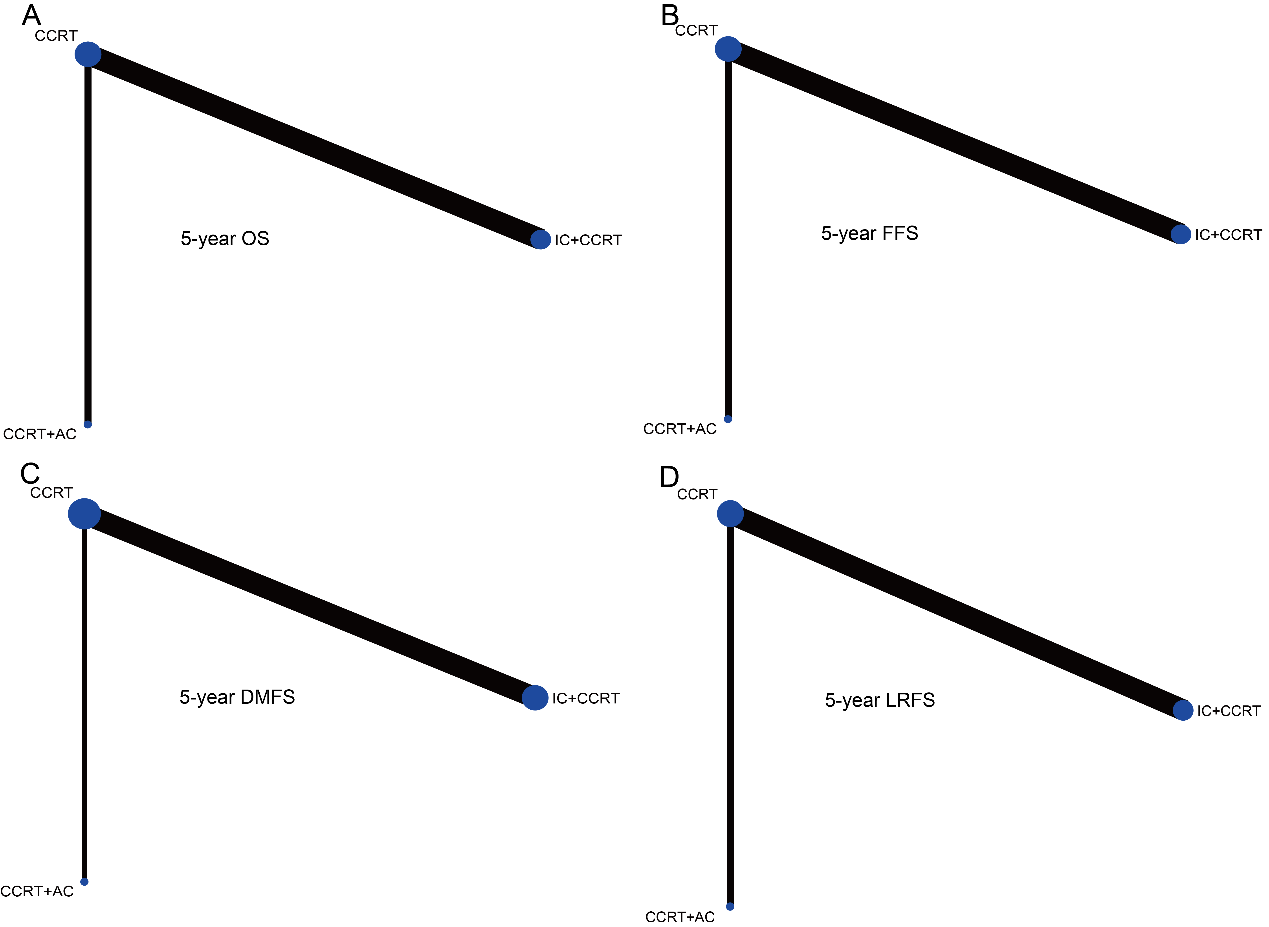


Figure S1 Network of the comparisons for the Bayesian network meta-analysis. Network for 5-year overall survival (A), failure-free survival (B), distant metastasis-free survival (C), and locoregional recurrence-free survival (D). each circular node represents a type of treatment. The dot size is proportional to the total number of patients who received a regimen. Each line represents a head-to-head comparison and the line width is proportional to the number of clinical trials comparing the connected treatment strategies.
