# Supplementary figures and images for: A Bayesian network meta-analysis of the primary definitive therapies for locoregionally advanced nasopharyngeal carcinoma: IC+CCRT, CCRT+AC, and CCRT alone
Source: PLoS One. 2022 Mar 18;17(3):e0265551. doi: 10.1371/journal.pone.0265551 (PMC8932567; doi:10.1371/journal.pone.0265551)

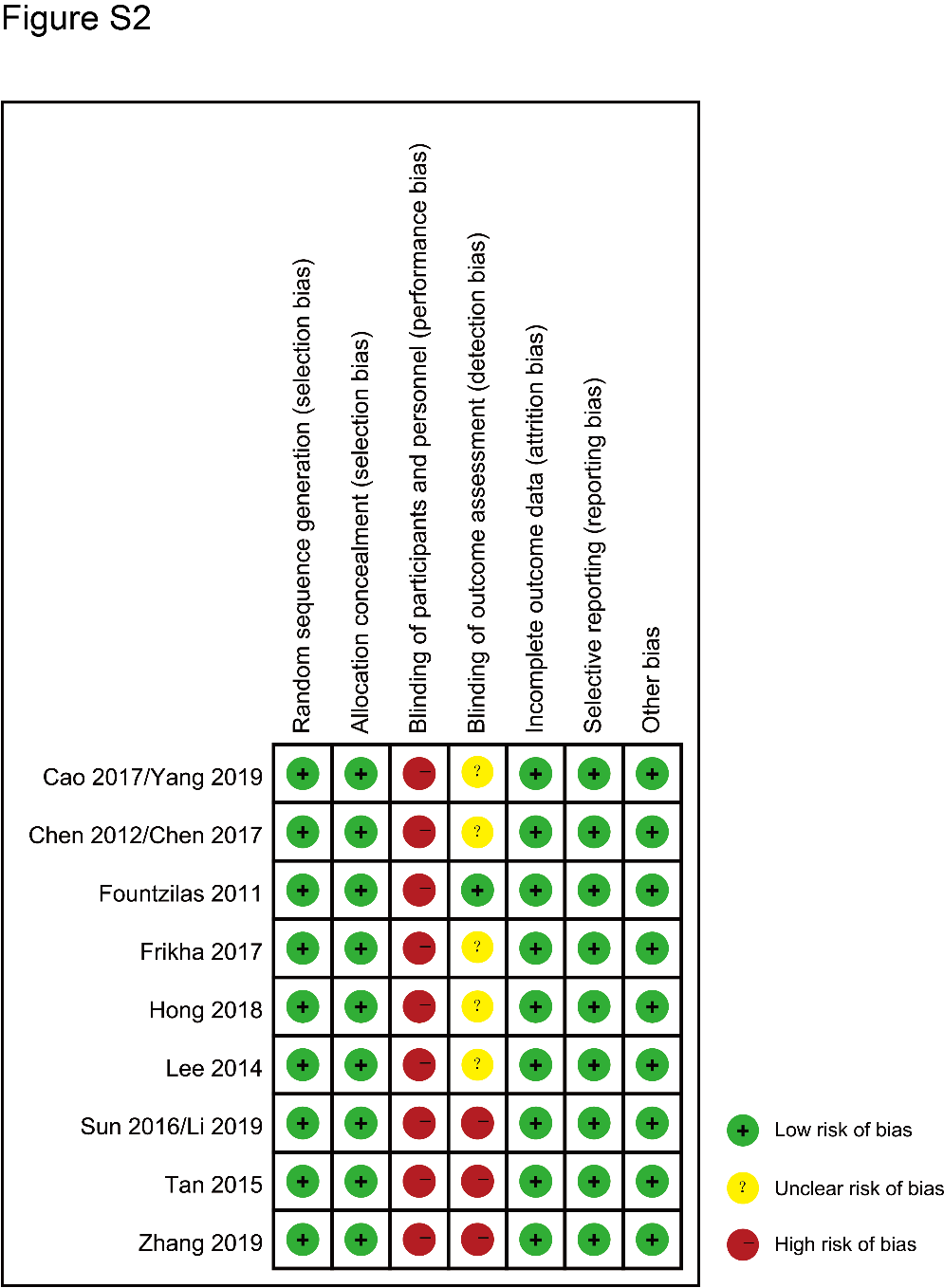
Figure S2 Risk of bias assessment in the analysis.

Supplement: S2 Fig — (DOCX) [file pone.0265551.s003.docx]
