# Supplementary material for: A Bayesian network meta-analysis of the primary definitive therapies for locoregionally advanced nasopharyngeal carcinoma: IC+CCRT, CCRT+AC, and CCRT alone
Source: PLoS One. 2022 Mar 18;17(3):e0265551. doi: 10.1371/journal.pone.0265551 (PMC8932567; doi:10.1371/journal.pone.0265551)
Supplement: S1 File — (DOCX) [file pone.0265551.s004.docx]

Search strategy in PubMed

1. induction OR neoadjuvant OR adjuvant

2. concurrent OR concomitant

3. cisplatin

4. chemotherapy OR chemoradiotherapy OR radiotherapy

5. nasopharyngeal

6. carcinoma OR cancer OR tumor

7. study OR trial

8. 1 and 2 and 3 and 4 and 5 and 6 and 7
